# Megavoltage radiotherapy effects on organs of the reticuloendothelial system

**DOI:** 10.1590/acb384123

**Published:** 2023-10-23

**Authors:** Fernando Pereira, Andy Petroianu, Jony Marques Geraldo, Claubia Pereira

**Affiliations:** 1 Universidade Federal de Minas Gerais – Departamento de Engenharia Nuclear – Belo Horizonte (MG) – Brazil.; 2 Universidade Federal de Minas Gerais – Departamento de Cirurgia – Belo Horizonte (MG) – Brazil.; 3 Hospital Luxemburgo – Centro de Radioterapia – Belo Horizonte (MG) – Brazil.

**Keywords:** Mononuclear Phagocyte System, Kupffer Cells, Radiotherapy

## Abstract

**Purpose:**

To study the uptake capacity of cells from the reticuloendothelial system after irradiation with high-energy X-rays.

**Methods:**

Eighteen male Wistar rats were distributed in three groups: group A (n = 6): control, unirradiated animals studied alongside animals from group B; group B (n = 6) and group C (n = 6): animals irradiated and studied after 24 and 48 hours, respectively. The rats were anesthetized and placed on a 10 MV linear accelerator. Next, they were irradiated in the abdominal region, with 8 Gy. Twenty-four (groups A and B) and 48 hours later (group C), a colloidal carbon solution (1 mL/kg) was intravenously injected in the tail vein. Fifty minutes later, the spleens and livers were withdrawn and prepared to be studied. Kupffer cells and splenic macrophages containing carbon pigments were counted in an optical microscope. Arithmetic means were calculated for each group and compared among them.

**Results:**

X-rays were associated with a reduced number of Kupffer cells containing colloidal carbon, proliferation and enlargement of biliary ducts, hypoplasia, and hepatocyte necrosis. In the irradiated spleen, the colloidal carbon uptake was concentrated in the marginal zone around the white pulp, with an inexpressive uptake of pigments by macrophages from white and red pulps.

**Conclusions:**

The X-rays in the rat abdomen are associated with a reduction in the Kupffer cells uptake of colloidal carbon, hepatocyte disorders, bile duct proliferation, and splenic uptake of colloidal carbon concentrated in the marginal zone.

## Introduction

Bone marrow pluripotent stem cells differentiate into monocytes, originating mononuclear phagocytic cells of the reticuloendothelial system (RES). In the blood stream, these cells carry diapedesis to different tissues of the RES, where they establish themselves as macrophages^1^.

The liver and spleen account for more than 80% of the RES and are the main organs responsible for the removal of abnormal cells, microorganisms, and foreign bodies from the blood stream. In these organs, what stand out are the Kupffer cells (KC), which represent nearly 40% of the hepatic cells, as well as the splenocytes–the spleen macrophages. RES is also responsible for hematopoiesis and the modulation of the immune system, as well as the regulation of the complement system^1^-^5^.

Radiotherapy interferes in the tumor microenvironment, provoking inflammatory responses in blood vessel walls, leading to severe lesions of endothelial cells. It also promotes the generation of MHC-I peptides involved in antigen-specific immune responses associated with the activation of cytotoxic T lymphocytes. Radiotherapy enhances phagocytosis of tumor cells by means of CD47 membrane receptor subexpression^6^. High-energy X-rays (XR) hinder the entrance of CD8+ lymphocytes into the tumor microenvironment and trigger the signaling of suppressive pathways, as well as enhance the radioresistance of regulatory T cells and suppressor cells derived from bone marrow^7^.

Previously, the same group of this work has published that the high-energy XR, used in radiotherapy, modifies the splenic clearance, enhancing the amount of marginal zone macrophages containing colloid particles^8^. Studies with technetium-phytate confirmed the splenic clearance modification in irradiated animals, revealing an enhancement of nearly 260% of the splenic uptake activity^9^. There was also reduction of the hepatic uptake activity of nearly 90%, which motivated the present histological investigation of the liver in view of potential radiation-induced damage to its parenchyma.

The XR influence on the RES remains not fully understood. Although the radiation-induced nitric oxide enhancement by tumor-associated M2 macrophages has already been described in the literature, knowledge on the radiotherapy influence on the macrophage uptake function of the RES is limited^7^. In this context, the purpose of this work was to study the *in-vivo* uptake capacity of cells from the RES after irradiation with high-energy XR.

## Methods

All animal experiments were complied with the ARRIVE guidelines and performed in accordance with the National Institutes of Health Guide for the Care and Use of Laboratory Animals. This project was authorized by the Ethics Committee on Research with Animals from the School of Medicine, Universidade Federal de Minas Gerais (Registry number: 115/2018).

Eighteen adults male Wistar rats, weighing 260 g, were accommodated in suitable cages at the School of Medicine’s Biotherium, receiving water and food *ad libitum*. The rats were separated into three groups (n = 6):

- Group A: unirradiated (control). After five hours of fasting, and under intramuscular anesthesia with 75 mg/kg of ketamine chloride associated with 6 mg/kg xylazine chloride^10^, 1 mL/kg of 50% colloidal carbon solution was administered into the tail vein. Fifty minutes later, the rats were euthanized with 225 mg/kg of ketamine chloride associated with 18 mg/kg xylazine chloride^10^. The spleen and liver were withdrawn and placed into a 10% formaldehyde solution for subsequent preparation in paraffin blocks and 4-micron thick histological sections. The tissue sections removed from different regions of the organs were then placed on glass slides and stained with hematoxylin and eosin to be studied;
- Groups B and C: rats irradiated and studied after 24 and 48 hours, respectively. The animals were prepared and anesthetized according to the protocol described for group A. They were irradiated with 8 Gy in the abdomen in a 10 MV linear accelerator. The isocenter was positioned at half the anteroposterior distance, and the irradiation with 8 Gy was performed with anteroposterior and posteroanterior incidences in two parallel and opposite fields, 4 × 5 cm each, with 4 Gy as the given dose in each field at the isocenter. Twenty-four later (group B) and 48 hours later (group C), a colloidal carbon solution was injected in the animals’ tail vein, and livers and spleens were removed from the animals 50 minutes later and prepared for microscopic studies, as established in the protocol described for group A.

**Figure 1:**
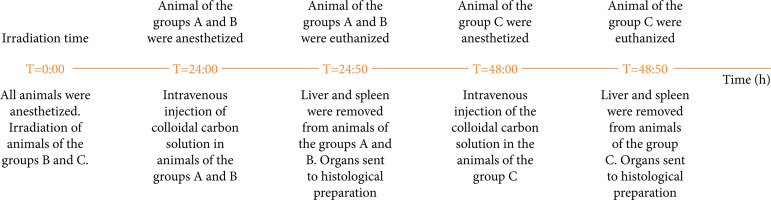
Description of the main steps of the experiment.

Source: Elaborated by the authors.

The colloidal carbon was obtained from China ink, which consists of charcoal powder mixed with Arabic gum and perfume. The ink was placed into a Becker flask and dried at 100°C. The residual paste was diluted at a proportion of 50 g/100 mL in sterile water^11^. Figure 1 summarizes the main steps of the experiment.

Two glass slides were prepared per animal, each one containing from five to seven sections of a single type of tissue. Thus, for each studied group of animals, a total of 36 glass slides were prepared, 18 for each type of tissue. The number of KC and phagocytizing splenocytes were counted in 10 consecutive microscopic fields randomly chosen on each glass slide, at a 400x magnification.

The arithmetic mean per group, along with its respective standard error of the mean, was calculated by summing the numbers obtained from all fields and dividing by the number of animals per group (N = 6). These values were compared among them using the one-way analysis of variance (ANOVA) technique and the post-hoc Tukey’s test. Differences corresponding to p < 0.05 were considered significant.

## Results

**Figure 2:**
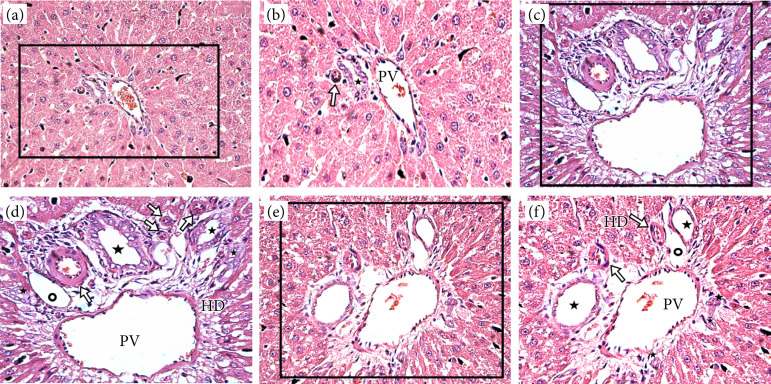
Morphological alterations in the portal spaces of animals irradiated with 8 Gy of X-rays. **(a)** Portal space in a hepatic tissue slice of unirradiated animal (group A) (hematoxylin and eosin, 400x). **(b)** Rows of normal hepatocytes, sinusoids, hepatic artery branches (arrow), portal vein, and bile ducts (star) (hematoxylin and eosin, zoom in the marked area of frame a). **(c)** Portal space in a hepatic tissue slice of irradiated animal (group B) (hematoxylin and eosin, 400x). **(d)** Enlarged portal vein, hydropic degeneration, and hepatocytes with central nucleus organized in a lace-like way. Increased amount of arterioles (arrow), enlargement and proliferation of bile ducts (*) and of lymphatic vessels (°) in comparison with portal space in unirradiated animal (frame b) (hematoxylin and eosin, zoom in the marked area of frame c); **(e)** Portal space in a hepatic tissue slice of irradiated animal (Group C) (hematoxylin and eosin, 400x). **(f)** Enlarged portal vein, hydropic degeneration, and hepatocytes with central nucleus organized in a lace-like way. Increased amount of arterioles (arrows), enlargement and proliferation of bile ducts (*) and of lymphatic vessels (°) in comparison with portal space in unirradiated animal (frame b) (hematoxylin and eosin, zoom in the marked area of frame e).

HD: hydropic degeneration; PV: portal vein. Source: Elaborated by the authors.

The animals tolerated the procedures satisfactorily. In all histological liver sections of the irradiated animals, hydropic degeneration and necrosis of hepatocytes were observed, especially in the perivascular region. The bile ducts were enlarged and replicated in both irradiated groups, with no apparent obstruction (Fig. 2).

**Figure 3:**
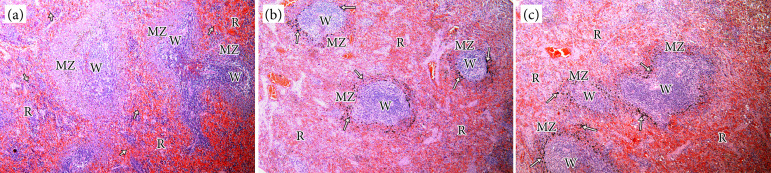
Colloidal carbon distribution in the splenic parenchyma of unirradiated animals and irradiated with 8 Gy of X-rays. **(a)** Group A: control, unirradiated spleen. Colloidal carbon particles injected into the blood and captured by macrophages (arrows) uniformly distributed in the white pulp, red pulp, and marginal zone (hematoxylin and eosin, 100x). **(b)** Group B: irradiated spleen studied 24 hours after irradiation. Colloidal carbon particles injected in the blood and captured by macrophages (arrows) mainly distributed in the marginal zone around the white pulp. Little colloidal carbon is observed inside macrophages of the red pulp (hematoxylin and eosin, 100x). **(c)** Group C: irradiated spleen studied 48 hours after irradiation. Colloidal carbon particles injected in the blood and captured by macrophages (arrows) mainly distributed in the marginal zone around the white pulp. Little colloidal carbon is observed inside macrophages of the red pulp (hematoxylin and eosin, 100x).

Source: elaborated by the authors. MZ: marginal zone; R: red pulp; W: white pulp.

The number of KCs with carbon pigments in their cytoplasm was reduced in the animals of both irradiated groups when compared to the control group (p < 0.05); however, no difference was observed between the irradiated groups (Table 1), which is in agreement with previous work with technetium-phytate^9^. The splenic macrophages containing carbon pigments were uniformly distributed in the splenic parenchyma of the control group. However, in the irradiated groups B and C, they were located mainly in the marginal zone around white pulps, as previously described^8^ (Fig. 3). No difference was found in the total number of splenic macrophages containing colloidal carbon among the three groups (Table 1).

**Table 1:** Number of splenic macrophages and Kupffer cells containing colloidal carbon (mean ± standard error of mean) of unirradiated and irradiated rats.

Groups	N	Splenic macrophages	Kupffer cells
A	6	419.17 ± 31.20	268.3 ± 13.0
B	6	412.35 ± 8.50	198.0 ± 19.8
C	6	428.29 ± 6.40	195.2 ± 15.5

N: number of animals per group; group A: unirradiated animals and injection of 1 mL/kg of 50 % colloidal carbon solution; groups B and C: animals irradiated in the abdominal region using two parallel and opposite radiation fields with 4 Gy in each, and injection of 1 mL/kg of 50% colloidal carbon solution 24 and 48 hours after irradiation, respectively. Source: Elaborated by the authors.

### Comparison among the mean numbers

- One-way ANOVA comparison among the mean number of splenic macrophages containing colloidal carbon from groups A, B and C: p was 0.82. The result was not significant at p < 0.05.
- One-way ANOVA comparison among the mean number of Kupffer cells containing colloidal carbon from groups A, B and C: p was 0.007. The result was significant at p < 0.05.
- Pairwise comparisons by post hoc Tukey’s test: between groups A and B, p was 0.02. The result was significant at p < 0.05; between groups A and C, p was 0.01. The result was significant at p < 0.05; between groups B and C, p was 0.99. The result was not significant at p < 0.05.

## Discussion

Since the 1980s, the uptake activity of the RES has been investigated by the authors by means of histological studies from experiments with colloidal elements and radiolabeled bacteria^12^-^14^. Nearly 90% of the colloidal carbon uptake was by splenic macrophages and KC^13^.

This study aimed to verify the megavoltage radiotherapy effects on RES, based on the previous results, which have shown that the concentration of the colloidal uptake activity in the splenic marginal zone after irradiation with a single dose of high-energy XR^8^, as well as a reduction of the hepatic uptake of a technetium-phytate solution injected in the animals^9^. The reduction of the hepatic uptake activity might be related with radiation-induced damage to the liver parenchyma that was histologically investigated in this work. Some of these effects have been known since 1920, first appearing in experimental studies with low-energy radiotherapy using colloids and bacteria^15^,^16^. Even that small radiation dose damaged tissues and influenced the RES activity^17^.

The depletion of the number of phagocytizing KC after irradiation, as verified in this study, differs from *in- vitro* experiments that used human macrophages and rodent monocytes irradiated with 10 Gy of XR^18^,^19^. In those studies, irradiation increased the inflammatory activity and the *Staphylococcus aureus* phagocytosis.

The enlargement and proliferation of the bile ducts associated with hepatocyte necrosis, resulting from portal space inflammatory reaction, as found in this work, have already been described in the literature^20^. Irradiation leads to smooth muscle fibers impairment that lose their tension, which is responsible for preserving the centrilobular vessels and the bile duct calibers. The fibers damaged by irradiation are unable to maintain the vessel and duct resistance to intraluminal tension. Consequently, dilation occurs.

In the presence of chronic inflammation due to radiotherapy, subsequent hepatic steatosis may be observed^21^. Connective tissue fibrosis, which can lead to sinusoid capillary stenosis, is also common under such adverse conditions^22^. Fractioned focal liver irradiation seems to be associated with bile duct cell replication with consequent ductal hyperplasia^23^. These events were observed in both groups B and C, that were studied 24 and 48 hours after irradiation. These findings confirms that non-fractioned radiotherapy is also able to trigger the replication of bile ducts.

Splenic macrophages can be found spread throughout the splenic parenchyma and are responsible for the removal of senescent red blood cells and other abnormal elements from the blood circulation. They produce immunoglobulins, complement factors, among others^12^. Considering that the white pulp is associated with the lymph node system, it is worth mentioning the possibility that radiotherapy immediately acts in these lymph nodes, as well as in other structures, including tonsils and intestinal Peyer patches^24^. By contrast, as shown in this work, megavoltage radiotherapy enhanced the clearance function of macrophages located in the splenic marginal zone.

## Conclusion

The high-energy XR in the rat abdomen are associated with a reduction in the KCs’ activity, hepatocytes disorders, enlargement and proliferation of bile duct, and splenic uptake activity concentrated in the marginal zone.

## Data Availability

All data sets were generated or analyzed in the current study
